# Metastatic meningeal carcinomatosis from lung cancer: Report of a rare case

**DOI:** 10.1111/1759-7714.13243

**Published:** 2019-11-17

**Authors:** Hui Liu, Li Liu, Xiaochen Zhang, Song Jin

**Affiliations:** ^1^ Department of Radiology Tianjin Huanhu Hospital Tianjin China

**Keywords:** Lung cancer, magnetic resonance imaging, meningeal carcinomatosis

## Abstract

Meningeal carcinomatosis (MC) refers to the diffuse or multifocal spread or infiltration of malignant tumors in the pia mater. It is a special distribution type of metastatic tumors in the central nervous system and one of the important reasons of death caused by metastatic malignant tumors. Here, we report a rare case of metastatic meningeal carcinomatosis from the lung cancer.

## Introduction

Lung cancer is the main cause of malignant tumor‐related death worldwide.^1^ Metastatic meningeal carcinomatosis is one of most serious complications of lung cancer.^2^ The incidence of meningeal metastasis in patients with non‐small cell lung cancer (NSCLC) ranges from 5%–15%, while the incidence of small cell lung cancer is even higher.^3^ The prognosis of meningeal carcinomatosis is poor with a median survival rate of less than six months. Patients with metastatic meningeal disease have a wide range of clinical symptoms including high intracranial pressure symptoms such as headache, nausea, vomiting, brain parenchyma and brain nerve involvement symptoms such as limb dysfunction, hearing loss, psychiatric symptoms and epileptic seizures. The diagnosis of meningeal metastasis is mainly based on the clinical manifestations, imaging and cerebrospinal fluid examination of the nervous system.^4^, ^5^ The gold diagnostic standard is still cerebrospinal fluid cytology examination. In our present case, we present a metastatic meningeal carcinomatosis from lung cancer.

## Case report

**Figure 1:**
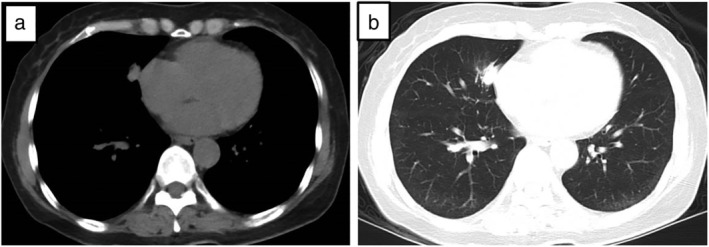
Chest CT scan demonstrated a right middle lobe space‐occupying lesion. **a**) Mediastinal window. (**b**) Lung window.

**Figure 2:**
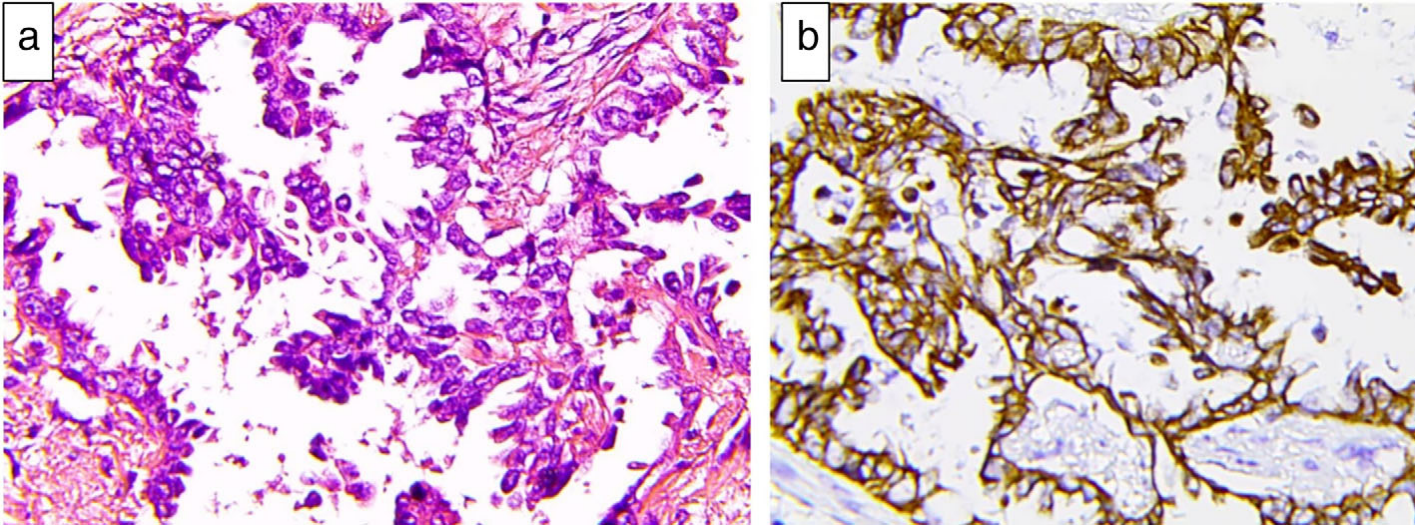
Pathology examination demonstrated lung adenocarcinoma. (**a**) H&E stain. (**b**) CK7 positive expression × 400.

**Figure 3:**
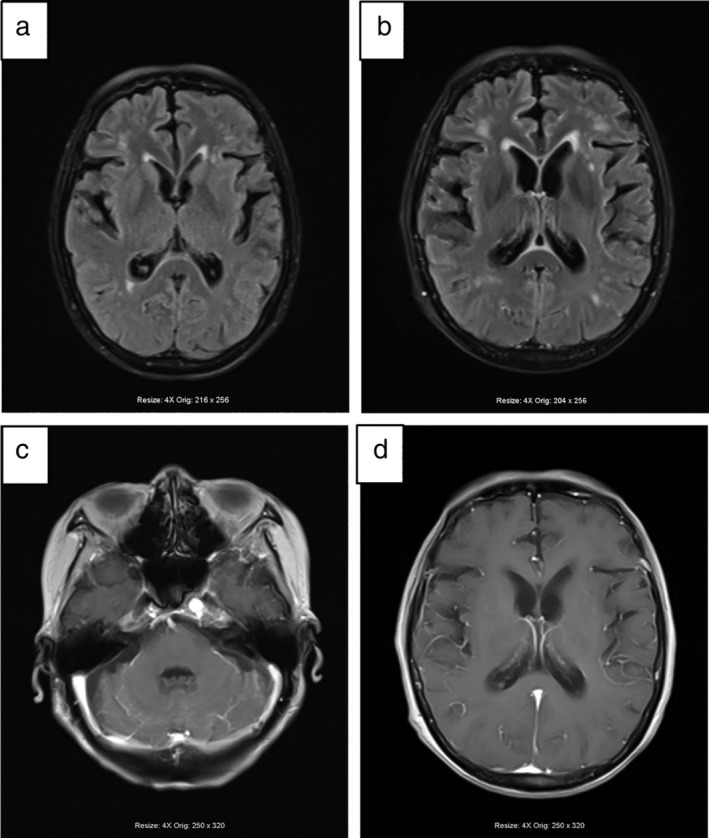
Brain MRI scan demonstrated metastatic meningeal carcinomatosis. (**a**) Axial fluid‐attenuated inversion recovery (FLAIR) image showed slightly hyperintense in the bilateral temporal‐parietal lobe sulcus. (**b**) Axial view of enhanced FLAIR: after intravenous injection of a contrast agent, abnormal linear enhancement changes were observed in the bilateral frontotemporal‐occipital sulcus. (**c**) Enhanced axial view of T1WI: abnormal linear contrast‐enhanced changes were observed in the ventral and dorsal medulla oblongata, bilateral cerebellar hemisphere plicate meninges. (**d**) Enhanced axial view of T1WI: abnormal linear contrast‐enhanced changes in the duodenal meninges in bilateral cerebral hemispheres were seen.

**Figure 4:**
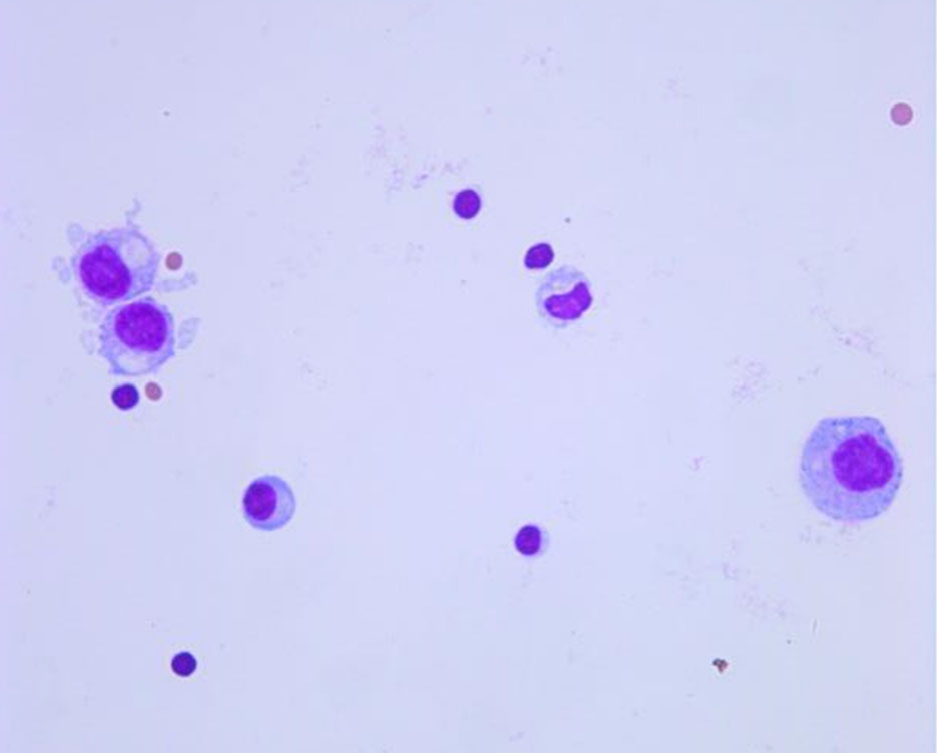
Nuclear heteromorphic cells were found in the cerebrospinal fluid of the patient.

A 61‐year‐old male was admitted to Tianjin Huanhu Hospital complaining of “intermittent dizziness, nausea and vomiting for half a month”. The patient had been diagnosed with a right middle lobe space‐occupying lesion 21 months previously and had undergone surgical treatment (Fig 1). Postoperative pathological examination of the right middle lobe space‐occupying lesion demonstrated lung adenocarcinoma (Fig 2). After admission to our hospital, enhanced cranial MRI examination was performed which indicated the presence of metastatic meningeal cancer (Fig 3). The cerebrospinal fluid of the patient was also examined and during the procedure a CSF pressure of 220 mm H2O was recorded. Results revealed a colorless clear liquid, 56 white blood cells/μL; sugar 1.02 mmol/L, chloride 128 mmol/L and it was protein positive. Heteromorphic cells were also detected in the cerebrospinal fluid (Fig 4). A diagnosis of metastatic meningeal carcinomatosis from lung cancer was therefore made.

## Discussion

Meningeal carcinomatosis can be caused by metastasis of tumors inside and outside the central nervous system. Among the primary tumors causing meningeal carcinomatosis, lung cancer is the most common malignant carcinoma, followed by gastric cancer, breast cancer, malignant lymphoma, malignant melanoma and pancreatic cancer. Hammerer *et al*. reported that approximately 10%–26% of lung cancer patients eventually developed meningeal metastasis disease.^6^ Adenocarcinoma of the lung or small cell lung cancer were the most common types of lung cancer that caused meningeal metastasis lesions.^7^ A diagnosis of meningeal metastasis is mainly based on the clinical manifestations, imaging and cerebrospinal fluid examination. In the present case, the patient developed metastatic meningeal carcinomatosis from the lung cancer, and the brain metastatic lesion was clearly demonstrated on MRI. Nuclear heteromorphic cells were also present in the cerebrospinal fluid of the patient.

According to study by Liu *et al*., there is no gold standard for diagnosing meningeal carcinomatosis, and no obvious specificity for the diagnosis of meningeal carcinomatosis by MRI.^8^ However, in our report, the abnormal enhancement of the meninges on contrast‐enhanced scanning was used as an important index for assisting with a diagnosis of meningeal carcinomatosis.
